# In vitro plant regeneration and *Agrobacterium*-mediated genetic transformation of a carnivorous plant, *Nepenthes mirabilis*

**DOI:** 10.1038/s41598-020-74108-7

**Published:** 2020-10-15

**Authors:** Sissi Miguel, Cindy Michel, Flore Biteau, Alain Hehn, Frédéric Bourgaud

**Affiliations:** 1 Plant Advanced Technologies SA, 54500 Vandœuvre-lès-Nancy, France; 2 Université de Lorraine – INRAE, LAE, 54000 Nancy, France

**Keywords:** Biotechnology, Plant sciences

## Abstract

In nutrient-poor habitats, carnivorous plants have developed novel feeding strategies based on the capture and digestion of prey and the assimilation of prey-derived nutrients by specialized traps. The *Nepenthes* genus, comprising nearly 160 species, presents a remarkable pitcher-shaped trap, leading to great interest among biologists, but the species of this genus are listed as threatened. In this work, we developed a protocol for reproducing *Nepenthes mirabilis* through shoot regeneration from calli. The cultivation of stem segments of *N. mirabilis* on MS medium containing thidiazuron induced organogenic calli after 10 weeks. Subcultured calli exposed to 6-benzylaminopurine showed shoot regeneration in 3 weeks with considerable yields (143 shoots/g of calli). Excised shoots transferred to medium with indole-3-butyric acid allowed rooting in 4 weeks, and rooted plantlets had a 100% survival rate. Based on this method, we also developed an *Agrobacterium*-mediated genetic transformation protocol using calli as explants and *ipt* as a positive method of selection. Twelve weeks post infection, regenerated shoots were observed at the surface of calli. Their transgenic status was confirmed by PCR and RT-PCR. In conclusion, this study provides an efficient method for regenerating *Nepenthes* and the first protocol for its stable genetic transformation, a new tool for studying carnivory.

## Introduction

Nearly 160 *Nepenthes* species are widespread in southwestern Asia, with the greatest diversity in Borneo and Sumatra^1–3^. These carnivorous plants have developed trap-shaped leaves that attract, capture, retain, and allow the digestion of prey such as arthropods. After being attracted by colorful peristomes, the floral scent, the flowers themselves or the fruit-like nectar, prey fall into the pitcher and are digested in a viscoelastic liquid containing hydrolytic enzymes^4–9^.

*Nepenthes* species are widely used in traditional medicine for their anti-osteoporotic, antimicrobial, and antioxidant properties^10–13^. Extracts prepared from these plants display hypoglycemic and hypolipidemic effects^14^ and can be used as eye drops for individuals with cataracts and night blindness^15^. Recent investigations described additional anti-inflammatory activities of methanolic extracts from *N. mirabilis*^16^, high antiproliferative effects of ethyl acetate extracts from *N. thorelii *× *(ventricosa *× *maxima)* on breast cancer cells^17^, and preferential antiproliferative action of ethyl acetate extracts from several *Nepenthes* hybrids against oral cancer cells^17–19^*.* These properties might be related to specialized metabolites such as naphthoquinones (i.e., plumbagin and its derivatives) (reviewed by Miguel, Hehn, and Bourgaud 2018). Several digestive enzymes have also been studied for different therapeutic applications, such as nepenthesins, which are considered an alternative source of enzymes for the treatment of celiac disease triggered by gluten proteins present in common grain products^20^. In addition to exploiting the natural characteristics of these species, recent studies have described the use of *Nepenthes* as a biotechnological tool for producing recombinant proteins that are secreted outside of plant tissues in pitchers. A proof-of-concept experiment was performed using a virus-based transient expression approach, leading to the production of 117 μg of GFP/m^2^ of soil area/year in pitcher secretions^21^. Consequently, a better understanding of outstanding proprieties linked at carnivory may be considered an important source of innovation.

Genetic transformation is a fundamental tool to study gene structure and function in plants and, in this context, features linked to carnivory. In the carnivorous plant area, only a few articles described stable transformation, and the Ti plasmid of *Agrobacterium tumefaciens* seems to be the most efficient. *Agrobacterium*-mediated transformation of *Drosera* has been reported and used to alter naphthoquinone content^22,23^ or to produce recombinant proteins^21^. *Agrobacterium*-mediated transformation of the aquatic carnivorous plant *Utricularia* has been reported recently^24^.

In the same way, we present in this work an easy-to-follow method for the plant regeneration and preliminary transformation efforts of *Nepenthes mirabilis* using *Agrobacterium tumefaciens*.

## Results

### Regeneration of *N. mirabilis* plants

#### Callus induction and maintenance

**Figure 1:**
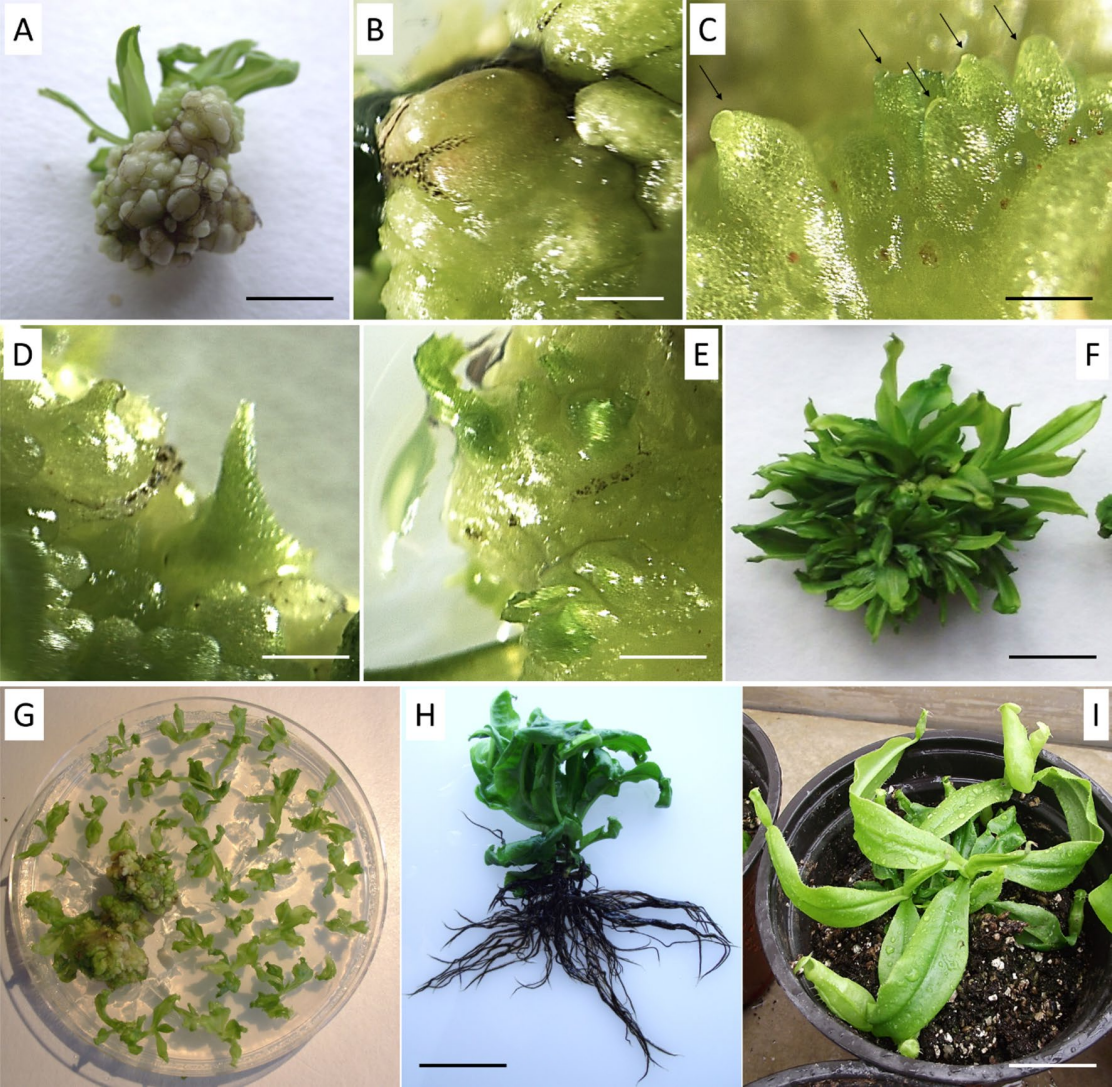
Indirect regeneration of *N. mirabilis*. (**A**) Organogenic calli from defoliated shoot fragments with a minimum of 2–3 nodes (bar = 0.5 cm); (**B**) surface of organogenic callus (bar = 1 mm); (**C**) formation of shoot apical meristems (indicated by black arrows) in the presence of 1 mg l^−1^ BAP in the medium (bar = 1 mm); (**D**) development of foliar primordia (bar = 1 mm); (**E**) development of shoots on the callus surface (bar = 1 mm); (**F**) calli after 10 weeks of exposure to BAP (bar = 1 cm); (**G**) multiple shoots separated from the calli after 10 weeks of exposure to BAP; (**H**) shoot rooted in the presence of 1 mg l^−1^ IBA after four months (bar = 1 cm); (**I**) acclimated plant 1 month after transfer to the greenhouse (bar = 2 cm).

Shoot fragments containing 2–3 nodes were collected from defoliated 6-month-old in vitro plants. To determine the best plant growth regulator combination leading to callus induction, different concentrations of TDZ (thidiazuron) and BAP (6-benzylaminopurine) were tested, either alone or in association with 2,4-d (2,4-dichlorophenoxyacetic acid) and kinetin (Table 1). The texture and the type of calli grown from nodes after 10 weeks were dependent on the type of growth regulators used. Friable calli were obtained exclusively in the presence of 2,4-D alone or associated with cytokinin. These friable calli turned brown within a few days when subcultured on the same medium. Yellow-white organogenic calli (Fig. 1A) with an epidermis covering nodular structures (Fig. 1B) were observed in the presence of different concentrations of cytokinins (30–84% efficiency, Table 1). The development of such calli was negatively impacted when auxins were added to the medium (Table 1). We highlight that TDZ, with a possible cytokinin-like effect, influenced the development of calli regardless of its concentration or its association with NAA (1-naphthaleneacetic acid) or 2,4-D. The effect of BAP was slightly different. We obtained evidence that the concentration was important when it was associated with 2,4-D but had no effect when associated with NAA (Table 1). The presence of cytokinins also impacted the maintenance of undifferentiated calli. TDZ allowed the establishment of a stable callus culture. It repressed shoot formation until at least 10 weeks after induction (Table 2) without altering the callus growth rate (multiplication of callus weight by 10.9- to 13.1-fold under different TDZ concentrations *versus* 11.15-fold without growth regulators). In contrast, in the presence of BAP, shoots appeared on the callus surface only a few weeks after induction.

**Table 1:** Effects of PGRs on organogenic callus induction from nodal stem segments after 10 weeks (% of callus induction).

0		Cytokinins (mg l^−1^)								
		0	TDZ				BAP			
			0.1	0.5	1	2	0.1	0.5	1	2
		0^a^	30^b^	51.90^b^	56.66^b^	84.44^b^	48.57^b^	56.67^b^	50.47^b^	48.33^b^
NAA	0.1	0^a^	51.11^b^	47.22^b^	52.38^b^	73.75^b^	8.89^a^	33.89^b^	50^b^	53.65^b^
	0.5	0^a^	62.30^b^	58.97^b^	39.52^b^	55.35^b^	3.33^a^	10^a, b, e^	30.55 ^b, e^	33.37^b^
	1	0^a^	21.42^d, f^	39.44^b, d^	18.09^a, b, d^	13.33^a, b, d^	0^a^	6.94^a, e^	6.11^a^	6.67^a^
	2	0^a^	4.76^a, f^	4.76^a, f^	11.11^a, f^	5.71^a^	0^a^	0^a^	0^a^	0^a^
2,4-D	0.1	2.78^a^	61.51^b^	44.64^b^	77.27^b^	63.80^b^	52.14^b^	58.33^b^	36.29^b^	40^b^
	0.5	3.33^a^	5.55^a^	5.79^a^	0^a^	7.77^a^	0^a^	2.38^a^	2.77^a^	5.55^a^
	1	0^a^	0^a^	0^a^	0^a^	0^a^	0^a^	5.55^a^	0^a^	0^a^
	2	0^a^	0^a^	0^a^	0^a^	0^a^	0^a^	0^a^	0^a^	0^a^

Ten explants were cultured in separated plates for each treatment. Experiments were repeated 3 times. Means followed by the same letters are not significantly different (*P* < 0.05).

**Table 2:** Effects of different concentrations of cytokinins on the regeneration of shoots from organogenic calli produced from nodal stem segments.

Growth regulators (mg l^−1^)	Regeneration efficiency (%)	Mean no. of shoots/g of calli
0	100.00^a^	51.81^a,d,g^
**TDZ**		
0.1	83.93^a,b^	35.48^a,d^
0.5	82.27^b^	40.00^a,b,e^
1	59.24^a,b^	16.32^b,c^
2	40.00^b,c^	7.52^c^
**BAP**		
0.1	96.97^a,c^	72.52^e,g^
0.5	96.66^a,c^	115.20^f,j,i^
1	100.00^a^	117.51^f,j,i^
2	96.67^a,c^	143.71^f^
**Kinetin**		
0.1	93.33^a,b,c^	83.28^g,j^
0.5	89.81^b,c^	119.20^i^
1	91.67^a,b,c^	91.32^g,i^
2	86.66^a,b,c^	48.52^h,d,e^

Ten callus fragments produced under the same growth regulator combination were cultured in separate plates for each treatment. Experiments were repeated three times. Means within a column followed by the same letters are not significantly different at the 5% level.

### Shoot regeneration

Some organogenic calli (200–400 mg) were subcultured on medium supplemented with BAP (0.1–2 mg l^−1^), kinetin (0.1–2 mg l^−1^) and TDZ (0.1–2 mg l^−1^) (Table 2). Three weeks after transfer to the regeneration medium, we observed that the surface of calli exposed to light became green and showed a granular appearance corresponding to shoot apical meristems (Fig. 1C), which later generated foliar primordia (Fig. 1D,E) in the presence of BAP and kinetin. The regeneration efficiency and the number of shoots per callus varied depending on the type and concentration of cytokinin used. Media devoid of growth regulators or supplemented with BAP (0.1–2 mg l^−1^) or kinetin (0.1–2 mg l^−1^) produced the highest frequency of shoot regeneration (87–100%, Table 2, Fig. 1F–G). Moreover, exposure to BAP (0.5–2 mg l^−1^) led to the regeneration of a considerable number of shoots (115–143 shoots/gr of calli, Table 2).

### Rooting

Shoots of 0.5–1 cm generated on 1 mg l^−1^ BAP medium were transferred to medium supplemented with various concentrations of IAA (indole-3-acetic acid), IBA (indole-3-butyric acid) and NAA (0.1–2 mg l^−1^) (Table 3). Root primordia were observed 4 weeks after transfer to the rooting medium. Regardless of the concentration that was used (0.1–2 mg l^−1^), IAA and IBA were clearly more efficient in promoting root induction, with success rates between 92 and 100% (Table 3). IBA at 0.5–2 mg l^−1^ allowed the development of a rooting system presenting a high number of roots (± 11 roots/shoot) with a size varying between 1.3 and 1.7 cm, divided into many ramifications (4–6 ramifications/root, Table 3, Fig. 1H). The plants grown on this medium also produced large aerial organs of between 5 and 7 cm. In contrast, plantlets rooted on the NAA medium grew more slowly because of a limited rooting system. Regenerated plantlets with well-developed roots were acclimated in a greenhouse with 100% efficiency for plants rooted in medium containing IBA (Fig. 1I).

**Table 3:** Effects of different concentrations of auxins on rooting efficiency and the morphology of the root system. Ten shoots regenerated under the same is the second key growth regulator combination were cultured in separate jars for each treatment. Experiments were repeated three times. Means within a column followed by the same letters are not significantly different at the 5% level.

Growth regulators (mg l^−1^)	Rooting efficiency (%)	No. of roots/shoot	Mean root length (cm)	Mean ramifications/root
0	89.25^a^	6.30^a,d^	1.41^a^	1.40^a,c^
**IAA**				
0.1	100.00^a^	7.84^a,b,f,h^	1.85^a^	1.96^a,b^
0.5	95.33^a^	9.10^b,c^	1.44^a^	2.95^b,f^
1	92.50^a^	8.86^b,c^	1.61^a^	1.43^a,c^
2	95.83^a^	9.86^b,c^	1.43^a^	1.09^c^
**IBA**				
0.1	91.67^a^	8.84^c,f^	1.43^a^	2.22^a,b^
0.5	100.00^a^	11.16^c^	1.69^a^	3.88^e,b^
1	100.00^a^	11.44^c,f^	1.48^a^	5.15^e,f^
2	96.66^a^	11.53^c^	1.37^a^	6.46^e^
**NAA**				
0.1	80.95^a^	9.61^b,c^	0.76^b^	1.78^b,c^
0.5	65.00^a^	5.46^d,h^	0.58^b^	1.20^a,c^
1	7.04^b^	0.25^e^	0.01^c^	0.00^g^
2	0.00^b^	^–^	^–^	^–^

### Genetic transformation and *ipt*-mediated positive selection

The establishment of an *Agrobacterium*-mediated transformation protocol requires several parameters to be set, such as the conditions for the selection of transformed cells. Preliminary experiments performed on *N. mirabilis* calli provided evidence that the necrosis of untransformed cells on selective medium (containing classical herbicides or antibiotics) led to the death of neighboring cells. We therefore switched to a positive selection system based on the production of isopentenyl transferase (ipt). This enzyme is reported to increase the production of endogenous cytokinins and lead to shoot formation.

**Figure 2:**
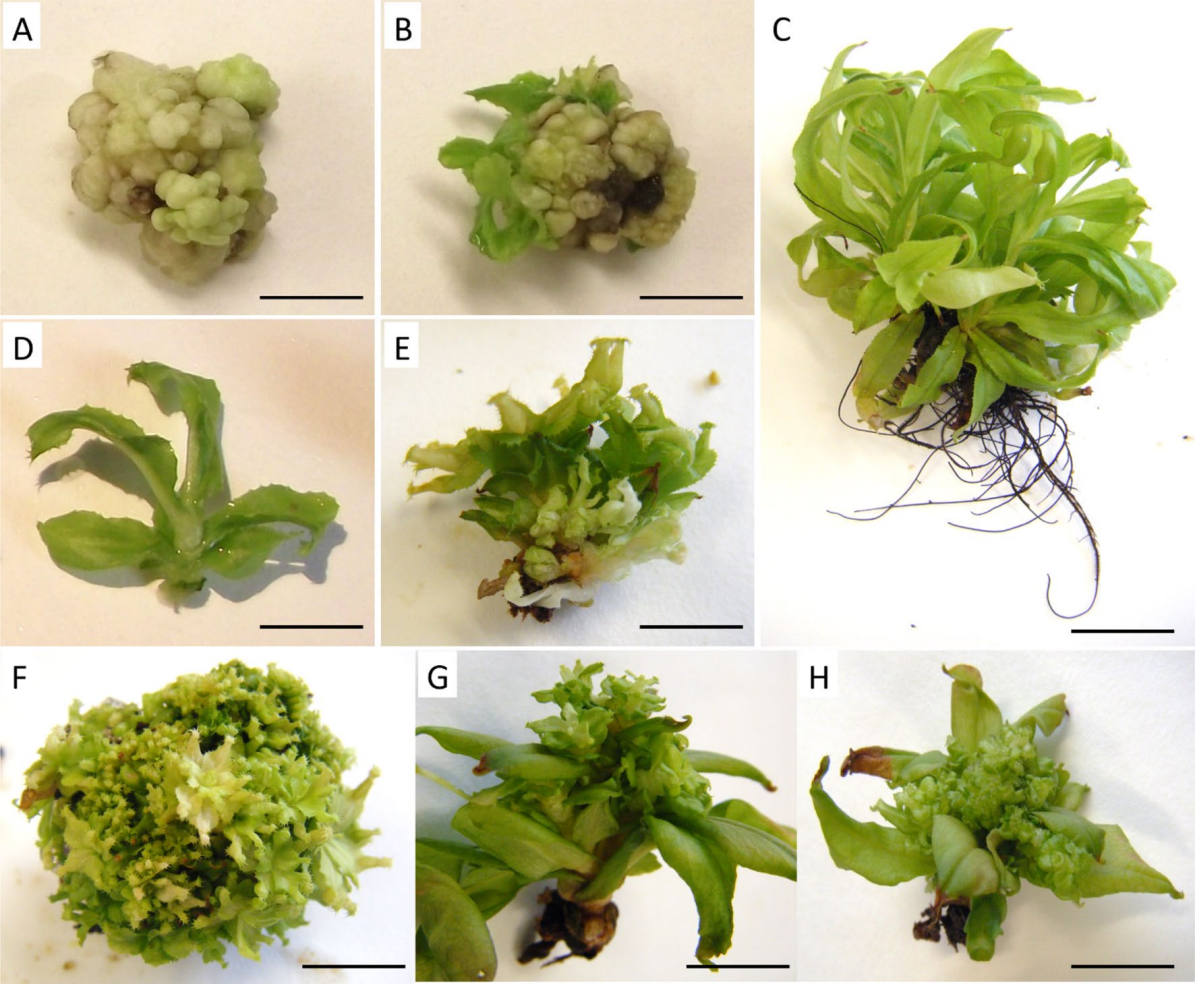
Genetic transformation of *N. mirabilis* by *A. tumefaciens*. (**A**) Calli infected by GV3101 agrobacteria (pGWB2-*mgfp5*) 10–12 weeks after transformation (bar = 1 cm); (**B**) calli infected by GV3101 agrobacteria (pGWB2-*ipt*) 10–12 weeks after transformation (bar = 1 cm); (**C**) wild-type plant regenerated through callus culture with 1 mg l^−1^ BAP and rooted in the presence of 1 mg l^−1^ IBA beginning at 6 months (bar = 2 cm); (**D**–**F**) evolution of an *ipt*-transgenic plantlet separated from calli (**D**) (bar = 0.5 cm) and exposed to 1 mg l^−1^ IBA beginning at 2 (**E**) (bar = 0.5 cm) and 6 months (**F**) (bar = 2 cm); (**G**,**H**) Morphology of *ipt*-transgenic plantlets obtained from different transformation events exposed to 1 mg l^−1^ IBA beginning at 6 months (bar = 2).

Undifferentiated callus fragments (200–400 mg) were used as explants for genetic transformation. Twenty-three series of transformation experiments including 20 calli each were conducted as described in the Materials and Methods section. The transformations were performed with *Agrobacterium* containing pGWB2-*ipt* or pGWB2-*mgfp5* as a negative control. To prevent shoot formation and maintain the cells in their undifferentiated state, calli dipped in an *Agrobacterium* suspension were cultured on medium supplemented with TDZ. In addition, low concentrations of hygromycin (1.5 mg l^−1^) were used to slow down the cell growth of nontransformed cells. Under these conditions, we assumed that the plantlets that appeared at the surface of the calli were able to express the *ipt* gene. Ten to twelve weeks postinfection, we observed spontaneously regenerated shoots at the surface of calli transformed with pGWB2-ipt (Fig. 2B), whereas no shoots were observed on any calli infected with *A. tumefaciens* harboring the pGWB2-*mgfp5* gene (Fig. 2A).

**Figure 3:**
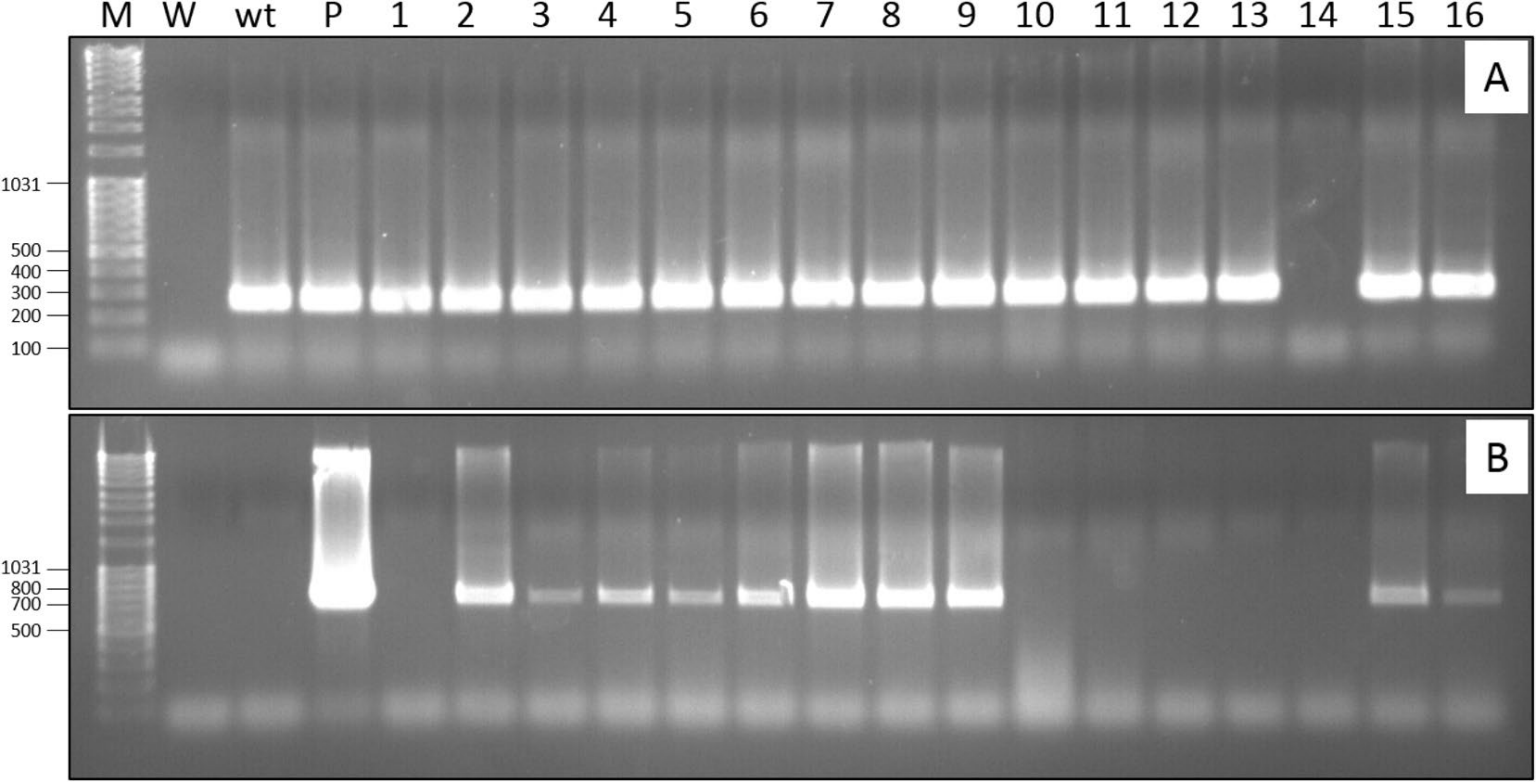
Molecular characterization of regenerated plants from different calli at 2–4 months after transformation. (**A**) PCR with primers specific to the Nepenthesin 2 gene as a housekeeping gene; (**B**) PCR with primers specific to the *ipt* gene; M: marker; W: water; wt: wild-type; P: plasmid; 1–16: transgenic plants derived from three separate series of genetic transformations.

To investigate the integration of the *ipt* gene in the genome, we collected a single shoot per callus and carried out specific PCR amplification of the transgene for each of them. The expected 720 bp-long amplicon was observed in 60.25% of the plants tested (Fig. 3B). The variation of the band intensity in comparison to the *Nepenthesin 2* gene (Fig. 3A) could, however, reflect a potential chimeric state of the plants.

**Figure 4:**
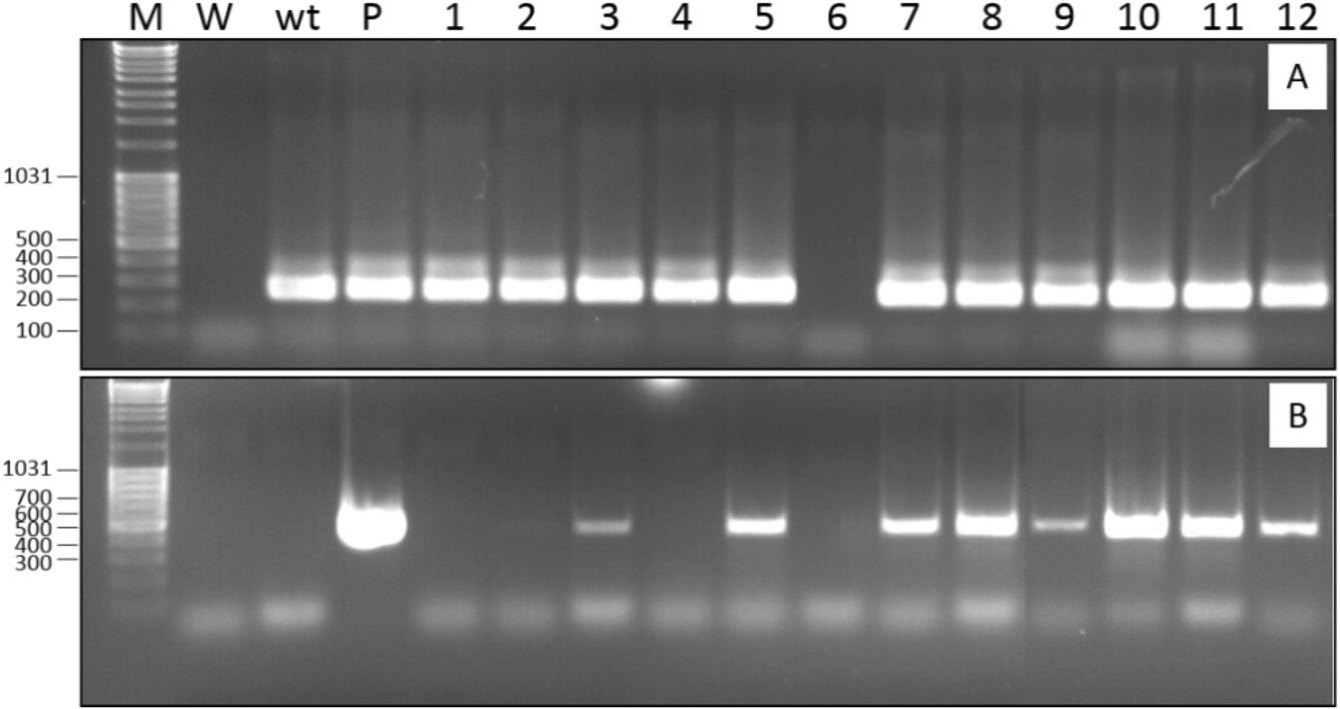
RT-PCR with RNA extracted from 6- to 8-month-old transformed plants. (**A**) RT-PCR with primers specific to the 18S ribosomal RNA gene as a housekeeping gene; (**B**) RT-PCR with primers specific to the *ipt* gene; M: marker; W: water; wt: wild-type; P: plasmid; 1–12: transgenic plants derived from nine independent series of genetic transformations.

The selected *ipt*-transformed plants further developed a typical cytokinin-overproducing phenotype (Fig. 2D–H) in comparison to wild-type plants (Fig. 2C). For several transformation events, the intensive reproduction of plantlets was observed at the crown level of shoots separated from calli. This led to the development of a pseudocallus that was entirely recovered by a large number of small plants displaying restrained stem elongation (Fig. 2D–F). For other plants, we observed reduced apical dominance and severe axillary branching at the apical and nodal levels, reflecting a typical phenotype observed in the presence of high concentrations of cytokinins (Fig. 2G,H). The rate of root initiation was extremely low for most transformation events despite exposure to 1 mg l^−1^ IBA for 18 months. This observation is consistent with the overproduction of endogenous cytokinins. Only 25 of 91 transgenic shoots developed adventitious roots, grew to the plantlet stage and were successfully acclimated. Reverse transcription-PCR experiments were performed on the transgenic plants to highlight the correlation between the expression of the *ipt* gene and the cytokinin-overproducing phenotype. We highlight the presence of a 430 bp-long amplicon corresponding to the expected *ipt* fragment of 6- to 8-month-old plants (Fig. 4). The intensity of the amplified DNA fragment could not be related to a specific phenotype. The differences between the 12 plants that we tested could therefore be related to the insertion site of the gene in the genome or the possible chimeric state of the transgenic plants.

## Discussion

In this work, we developed a method for regenerating and genetically transforming *Nepenthes mirabilis* under in vitro conditions. In the first part of the study, we identified a cocktail of growth regulators based on TDZ, BAP and IBA necessary for successful plant regeneration through indirect organogenesis allowing the regeneration of up to + /− 140 shoots/g of calli.

Callus cultures could be established from multinodal stem tissues exposed to different concentrations of TDZ with a 30–84% rate of success. This plant growth regulator (PGR) allows calli to be maintained in their undifferentiated state by inhibiting shoot regeneration. TDZ seems to have the ability to switch the internal hormonal balance from nodal meristem production toward a simple cell proliferation program. More than 800 articles report the use of TDZ as a cytokinin-like PGR that has been widely used for the induction of plant regeneration^25^. The potency of TDZ has been demonstrated for the *in vitro* propagation of many recalcitrant, woody and legume species. For example, it has been used for the culture of *Echinacea purpurea* L.^25^, *Vitex trifolia* L.^26^, *Curcuma longa* L.^27^, *Bauhinia tomentosa* L.^28^ and *Psoralea corylifolia*^29^. In contrast to other cytokines, TDZ is resistant to endogenous cytokinin oxidases and is therefore rather stable in tissue culture. It has also been shown that it can inhibit the activity of these enzymes, resulting in the accumulation of endogenous purine cytokinins (reviewed by Dewir et al.^30^). Finally, TDZ was reported to maintain stable callus culture since it does not alter the growth rate and shoot formation is repressed at least until 10 weeks after induction. Dewir et al. indicated that plant tissues exposed to TDZ for a long duration are subjected to an overdose, resulting in the inhibition of shoot proliferation^30^.

BAP is the second key growth regulator that plays an important role in the method we developed. This molecule is reported to be used for the activation of seed germination with an efficiency of 26–97%^31,32^. In addition to this property, this growth regulator causes an increase in the development of shoot apical meristems on callus surfaces when added to the culture medium (0.5–2 mg l^−1^). This observation is consistent with many reports concerning the in vitro culture of other *Nepenthes* species*.* Indeed, BAP seems to be one of the most efficient cytokinins for the generation of shoots from nodal stem fragments or the apical region. The success of this induction method is reported to be between 90 and 95%^33–36^. We estimate that the ability of BAP to activate meristems and regenerate several shoots from the same node leads to a 2.5-fold increase in shoot apical meristems on the callus surface in comparison to culture conditions without growth regulator.

Finally, the third growth regulator necessary in our culture method is IBA. When shoots were cultured on medium containing 0.5–2 mg l^−1^ IBA, we observed nearly 100% rooting. This PGR allows the generation of multiple large primary roots displaying substantial ramification.

Today, 122 *Nepenthes* species are included in the International Union for Conservation of Nature (IUCN) red list of threatened species. Among these species, 14 are classified as endangered, and 10 exhibit a critical status. One way to save these plants is to reproduce them in vitro. Several relevant reports are already available. In vitro reproduction for the large-scale propagation of *N. khasiana*, *N. mirabilis* and *N. macfarlanei* has been performed by seed germination and tissue culture^31–35^. Germination under in vitro conditions is difficult and depends on the freshness of seeds and their contamination state. Therefore, micropropagation from nodes is mostly described in the literature as an interesting option for rapid mass reproduction and large-scale propagation. The method we describe in this report seems to be an interesting approach for efficiently reproducing *N. mirabilis*. It could be considered for application to other endangered *Nepenthes* species for their conservation, although it might be necessary to determine the appropriate PRG concentrations for each of them. Although the approach is very efficient and fast, side effects such as somaclonal variations should potentially be considered. Such modifications were observed by Devi and collaborators for *N. khasiana*. These authors highlighted cytological variations in three consecutive regenerations of *N. khasiana* from nodal stem fragments^37,38^. Since TDZ can cause abnormalities in plant tissue cultures, such an evaluation should be performed under our method.

In the second part of our study, we set up an *Agrobacterium-*mediated genetic transformation protocol for *Nepenthes mirabilis*. Transgenic plants were obtained from calli using a positive selection strategy based on the expression of isopentenyl transferase. We observed that when we used this approach, nearly 60% of the regenerated shoots were transgenic. Such a strategy might therefore be considered for gene functional analysis purposes, and any gene of interest might be included in the T-DNA of a binary vector under the control of the P35S promoter containing the *ipt* gene. This efficient method applied in *N. mirabilis* was previously used to generate luciferase- or *gus*-transgenic tobacco plants^39,40^. Although this method is efficient, it could still be improved. For example, our PCR analyses seem to highlight chimerism events in the transgenic plants regenerated from callus culture despite the inclusion of hygromycin in the medium. Ebinuma and colleagues demonstrated that *ipt* under the control of the P35S promoter leads to cytokinin overproduction in transformed cells. The hormone likely diffuses to wild-type neighboring cells and induces the generation of chimeric plants or wild-type shoots near transgenic shoots^41^. Kunkel et al. (1999) proposed that the number of nontransgenic regenerants could be reduced by exposing tissue to high ratios of auxins favorable to root regeneration and suppressing shoot regeneration to counterbalance cytokinin effects^39^. Such a strategy could be applied to *N. mirabilis* calli after the transformation step.

Another drawback of this strategy is the generation of typical detrimental phenotypes related to cytokinin overproduction, characterized by strongly restrained root initiation and shoot elongation. Therefore, most of the *ipt*-transformed shoots could not produce roots in the rooting medium and could not be acclimatized. However, several solutions have been described in the literature to limit this negative effect. The first consists of fusing the *ipt* gene with an inducible promoter, such as a copper-^42^, ethanol-^43^, tetracycline-^44,45^ or dexamethasone (Dex)-inducible promoter^39^. Another possibility might be to remove the *ipt* gene using the MAT (multi-auto-transformation) vector system. This system allows the excision of the *ipt* gene though site-specific recombination induced by a recombinase of the R/RS system^41,46,47^. This approach has been successfully used in several plant species, such as tobacco^41,41^, hybrid aspens^48^, rice^49^, *Nierembergia*^40^, apricot^50^, white poplar^51^, citrus^52^, cassava^53^, *Kalanchoe blossfeldiana*^54^, petunia^55^, potato^56^ and tomato^57^. An alternative method relying on the Cre/loxP site-recombination system also allows the generation of marker-free transgenic plants, as described for citrus^58^.

The study described in this article provides some new perspectives related to the preservation of *Nepenthes* species. The efficient in vitro method that we developed provides an interesting tool for reproducing these plants and subsequently contributing to their restoration in the context of a general decrease in biodiversity. The work also provides a new tool for investigating the molecular mechanisms involved in plant carnivory, such as those related to the formation of the fascinating traps of these plants. For example, overexpression or genomic editing methods might help to provide physiological validation of genes involved in pitcher development, such as the recently described ASYMMETRIC LEAVES1 and REVOLUTA genes^59^. Genetic and proteomic resources have greatly increased over the last 10 years, including a multitude of RNA-seq and genome libraries from different *Nepenthes* species. All of these resources available in public databases can now be fully utilized for understanding how these plants originated and evolved.

## Materials and methods

### Plant material and tissue culture

In vitro* N. mirabilis* cultures were established from fresh seeds supplied by Districarnivores (www.districarnivores.com). Seeds were sterilized by total immersion in a diluted commercial bleach solution containing 0.25% sodium hypochlorite for 5 min and washed three times with sterile water. After a drying step on sterile paper, the seeds were sown in Petri dishes of 58 mm in diameter and 15 mm in depth on ¼ Murashige and Skoog (MS) medium (Murashige, 1962) containing a twofold MS vitamin mixture, 2% (w/v) sucrose, 0.05% (w/v) casein hydrolysate, 0.07% (w/v) 2-(N-morpholino)ethanesulfonic acid, 6 mg/L ProClin 200 (Sigma-Aldrich, St Louis, Mo, USA) and 0.7% (w/v) HP696 agar (Kalys, Bernin, France). The seeds were incubated under a 16 h/8 h day/night photoperiod provided by natural white fluorescent lamps at a temperature of 23 °C. The seedlings were then transferred to ½ MS medium containing a twofold MS vitamin mixture, 2% (w/v) sucrose, 0.05% (w/v) casein hydrolysate, 0.07% (w/v) 2-(N-morpholino)ethanesulfonic acid and 0.7% (w/v) HP696 agar (Kalys, Bernin, France). The pH of all media was adjusted to 5.8 before autoclaving.

Nodal shoot fragments with a minimum of 2–3 nodes were exposed to solidified basal medium (BM) composed of ¼ MS medium containing a twofold MS vitamin mixture, 2% (w/v) sucrose, 0.05% (w/v) casein hydrolysate, 0.07% (w/v) 2-(N-morpholino)ethanesulfonic acid and 0.7% (w/v) HP696 agar supplemented with different auxins, such as 2,4-D (0.1, 0.5, 1 and 2 mg/L) and NAA (0.1, 0.5, 1 and 2 mg/L), either alone or associated with a cytokinin, such as TDZ (0.1, 0.5, 1 or 2 mg/L) or BAP (0.1, 0.5, 1 or 2 mg/L). The Petri dishes were incubated in a growth chamber under a 16 h/8 h day/night photoperiod provided by natural white fluorescent lamps at a temperature of 23 °C. After callus induction, explants were transferred to BM either without growth regulators or supplemented with 0.1, 0.5, 1 or 2 mg/L TDZ, BAP or kinetin for adventitious shoot induction. For root induction, the shoots were cut and transferred to BM supplemented with 0.1, 0.5, 1 or 2 mg/L NAA, IAA or IBA. Finally, the obtained plantlets were acclimatized in a 1:1 mixture of peat and vermiculite in heated greenhouses with natural light at a temperature of 23 °C and humidity of 75–85%.

### Preparation of *A. tumefaciens*

**Figure 5:**
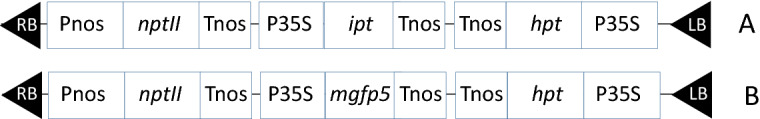
Schematic representation of the T-DNA used in this study, pGWB2-*ipt* (**A**) and pGWB2-*gfp* (**B**). RB: right border; LB: left border; Tnos: terminator of nopaline synthase gene; Pnos: promotor of nopaline synthase gene; P35S: CaMV 35S promoter; *nptII*: neomycin phosphotransferase gene; *hpt*: hygromycin phosphotransferase; *mgfp5*: green fluorescent protein gene; *ipt*: isopentenyl transferase gene.

The *ipt* gene (GenBank ID: DQ058764.1) was synthetized and cloned into the pUC plasmid by Thermo Fisher Scientific. The *m-gfp5-*ER gene came from pBin-*m-gfp5-*ER, provided by Pr Haselhoff (Cambridge University, UK). *ipt* and *m-gfp5-*ER were amplified with Platinium *Taq* DNA Polymerase High Fidelity (Invitrogen) using ipt.cp1_F (5′-TCGACATGGATCTACGTCTAATTTTCGG-3′) and ipt.cp1_R (5′-AGCTCTCACATTCGAAATGGTGGTCC-3′) for ipt or using gfp.pBin_F (5′-GGATCCAAGGAGATATAACAATGAAGACTAATCTTTTTCTC-3′) and gfp.pBin_R (5′-GAGCTCTTAAAGCTCATCATGTTTGTATAGTTCATCC-3′) as primers. The PCR products were introduced into the pCR8 plasmid using the pCR8/GW/TOPO TA cloning system (Invitrogen). The genes of interest were further integrated into the pGWB2-GW plasmid^60^ using the LR recombination system (Gateway LR Clonase II Enzyme Mix; Invitrogen) (Fig. 5).

The *A. tumefaciens* GV310I strain transformed with the recombinant pGWB2-*ipt* or pGWB2-m-*gfp5-ER* plasmid was cultured in liquid YEB medium containing 30 mg/l rifampicin, 30 mg/l gentamycin and 30 mg/l kanamycin at 28 °C for 2 days at 120 rpm. Four hours before plant transformation, 100 µM acetosyringone was added to the bacterial cultures from a − 20 °C-stored stock solution of 1 M in concentration. The bacteria were pelleted by centrifugation for 12 min at 3500×*g* and resuspended at a cell density of OD_600_ 0.7 ± 0.1 in liquid BM.

### Genetic transformation of *N. mirabilis*

Undifferentiated callus fragments (200–400 mg) were dipped in an *A. tumefaciens* culture for 15 min, briefly dried on sterile filter paper and transferred to solidified BM supplemented with 1 mg l^−1^ TDZ. After 3 days of cocultivation (16 h/8 h day/night photoperiod at a temperature of 23 °C), the calli were transferred to BM supplemented with 1 mg l^−1^ TDZ, 1.5 mg l^−1^ hygromycin and 200 mg l^−1^ cefotaxime. To favor rooting, the regenerated plantlets were separated from the calli and transferred to BM supplemented with 1 mg l^−1^ IBA, 1.5 mg l^−1^ hygromycin and 200 mg l^−1^ cefotaxime.

### PCR-based molecular characterization of the transgenic plants

PCR amplification was conducted from leaf pieces collected from plantlets using the Phire Plant Direct PCR kit (FINNZYMES). The quality of the PCR amplification was assessed using specific primers targeting the *Nepenthesin 2* gene (GenBank ID: AB114915.1) (Nep2_F: 5′-GGCCTCACCACTATACTCTGTGGTACTTGGC-3′ and Nep2_R: 5′-CGAGATCAACACGAAGGCCGGGTTGTGG-3′). The primers designed to amplify *ipt* were ipt.cp1_F and ipt.cp1_R.

### Reverse transcription-PCR analyses

Total RNA was extracted from transgenic plants using the Spectrum Plant Total RNA kit (Sigma-Aldrich, St Louis, Mo, USA) according to the manufacturer’s instructions. To efficiently remove genomic DNA, RNA was digested with the Turbo DNA Free Kit (AM1907M, Ambion, Thermo Fisher Scientific, Waltham, Mass, USA).

RT-PCR experiments were performed with 50 ng of RNA using the SuperScriptIII RT-PCR One-Step system (Thermo Fischer Scientific, Waltham, MA, USA). We used primers specific to the 18S ribosomal RNA gene (GenBank ID: KM393198.1) (18SrRNA_F: 5′-CTGCGGCTTAATTTGACTCAACACGGG-3′ and 18SrRNA_R: 5′-CAGACCTGTTATTGCCTCAAACTTCCG-3′) for housekeeping gene amplification. The primers designed to amplify *ipt* were ipt.cp2_F: 5′-GGGTATCATTACAGCCAAGCAAGCTCATG-3′ and ipt.cp2_R: 5′-CGACGCGCATGGATTAGAAACTCCTG-3′.

### Statistical analysis

For the establishment of regeneration tests, the experiments were performed under a randomized design. Ten explants were cultured in separated plates for each treatment. Experiments were repeated 3 times. The data collected were subjected to Student’s t-tests (*P* ≤ 0.05).
